# Why Do the Cosmic Rays Induce Aging?

**DOI:** 10.3389/fphys.2020.00955

**Published:** 2020-08-12

**Authors:** Anna Giovanetti, Flavia Tortolici, Stefano Rufini

**Affiliations:** ^1^ENEA, Department of Energy and Sustainable Economic, Rome, Italy; ^2^Department of Biology, University of Rome Tor Vergata, Rome, Italy

**Keywords:** aging, cosmic rays, mitochondrion, DNA damage, cell signaling

## Abstract

The increasing duration of space missions involves a progressively higher exposure of astronauts to cosmic rays, whose most hazardous component is made up of High-Atomic number and High-Energy (HZE) ions. HZE ions interact along their tracks with biological molecules inducing changes on living material qualitatively different from that observed after irradiation for therapeutic purposes or following nuclear accidents. HZE ions trigger in cells different responses initialized by DNA damage and mitochondria dysregulation, which cause a prolonged state of sterile inflammation in the tissues. These cellular phenomena may explain why spending time in space was found to cause the onset of a series of diseases normally related to aging. These changes that mimic aging but take place more quickly make space flights also an opportunity to study the mechanisms underlying aging. In this short review, we describe the biological mechanisms underlying cell senescence and aging; the peculiar characteristics of HZE ions, their interaction with living matter and the effects on the organism; the key role of mitochondria in HZE ion-induced health effects and aging-related phenomena.

## Introduction

In the hypothesis of exploration and settlement on the other planets, it is necessary to make long-duration space flights safer for astronauts. A detrimental effect often observed is indeed a state of frailty such as cardiovascular and cognitive disorders. The presence of these typical features of aging is very intriguing, and the cellular pathways involved in both cases are investigated not only to minimize the health risk of space missions but also to bring insight on the age-related mechanisms. While being aware that astronauts are subjected to numerous stress factors, this minireview is concerning the effects induced by the heavy ions’ component of cosmic rays, that thanks to their high linear energy transfer (LET), they are able to induce biological effects that persist over time and are transmitted through the tissue.

We will focus mainly on the triggered oxidative imbalance and DNA damage that represent the common elements underlying both the detrimental effects induced by cosmic rays and the aging process.

## Aging: Definition and Characteristics

Aging is an inevitable decay due to the passage of time that involves a general progressive loss of homeostasis in various organs and in the whole body, causing the impairment of vital functions and increased vulnerability to death. The loss of homeostasis leads, sooner or later, to the development of pathologies typical, such as diabetes, cancer, cardiovascular diseases, and alteration of cognitive functions (Khan et al., 2017).

In accordance with López-Otín et al. (2013), there are three families of distinctive signs of aging: (i) genomic instability caused by the deterioration of telomeres, epigenetic alterations, and loss of proteostasis; (ii) the accentuation of the compensatory responses, first of all the increase in apoptosis, cell senescence, and mitochondrial imbalance; (iii) finally, the exhaustion of stem cells and alteration in intercellular communication.

The theory that the accumulation of stochastic damage to DNA is the initial cause of almost all the listed aging processes is supported by various results (Niedernhofer et al., 2018). To avoid the onset of neoplasia, some of the DNA damage response (DDR) mechanisms, different according to the damage, intervene for the repair. In case damage is not repairable early, cells are initiated toward a programmed death or forced into a state of replicative quiescence typical of senescent cells. A close correlation between aging and persistence of unrepaired DNA damage is demonstrated by the observation that patients bearing DNA repair genetic defects such as xeroderma pigmentosum, ataxia telangiectasia, etc., show the hallmarks of premature aging (Kraemer and DiGiovanna, 2015). Likewise, exposure to environmental or therapeutic genotoxic compounds induces the early onset of aging features (Darby et al., 2013). The persistence of chromosomal damage is caused by reactive oxygen species (ROS) originated both by environmental factors such as radiation or mitochondrial imbalance (Sohal et al., 1994). In fact, the DNA damage from ionizing radiation has thus been attributed to the formation of radicals from the radiolysis of water molecules, as will be treated extensively in a later paragraph. More recently, also in light of the progress on understanding radicals as elements of intracellular and intercellular communication (Paladino et al., 2018), more and more weight is being given to biological pathways mediated by mitochondrial oxide-reduction while intermediate metabolic products can lead to oxidation, nitrosylation, and alkylation of DNA (Hekimi et al., 2011). Whatever their origin, ROS are able to induce DNA damage either directly or indirectly by interfering with the activity of the epigenetic regulatory factors and the DDR proteins. The most harmful DNA oxidation product directly induced by ROS imbalance is the 7,8-dihydro-8-oxoguanine (8-oxoG) lesion, the level of which correlates with age-related diseases such as cardiovascular diseases and neurodegeneration (Jacob et al., 2013). Silencing the OGG1 gene, which codes for the enzyme responsible for the removal of 8-oxoG, induced a massive amplification of oxidized DNA (Larsen et al., 2006), thus perpetuating chromosomal damage.

DNA repair capacity was found to decrease with aging (Imam et al., 2006); this, together with the increase in ROS production, causes an increased persistency. In many cases, the presence of unrepaired chromosomal damage does not trigger the mechanisms leading to programmed death but rather to senescence. All these processes seem to involve a high number of regulatory proteins, among which a pivotal role is played by p53 and p21, which, through the inhibition of some cyclin kinases, inhibit apoptosis and keep the cell in a replicative stasis (Regulski, 2017).

One of the characteristics of those senescent cells in which unrepaired chromosomal foci persist is the capacity to “spread” harmful information to healthy neighboring cells, increasing in that way the tissue’s number of senescent cells. This phenomenon, generically called bystander effect, passes through the release by the secretory senescent cells [senescence-associated secretory phenotype (SASP)] of a complex secretome, which includes cytokines, growth factors, and microvesicles (Acosta et al., 2013; Urbanelli et al., 2016). The physiological aim of this process may be to “predispose” the tissue cells to respond to subsequent stress, avoiding the formation of neoplasia. The accumulation of senescent cells or their loss for apoptosis results in a progressive decrease of tissue functionality that is a typical feature of aging. According to this interpretation, aging would be nothing more than the consequences on cells of the signals triggered by the persistence of chromosomal damage and by the oxidative imbalance.

We will now explore how ionizing radiation in general but cosmic rays in particular may trigger this process faster by virtue of their peculiarities.

## Cosmic Rays Interaction With Biological Matter

During long space missions, astronauts are subjected to a particularly harsh environment due to multiple factors as isolation, microgravity and macrogravity, and galactic cosmic rays (GCRs) and solar particle events (SPEs). While on Earth, the main component of natural radiation consists of radon, its decay products, and gamma rays from the decay of natural radioisotopes. GCRs contain a dynamic mixture of hydrogen (H) and helium (He) nuclei, and High-Atomic number and High-Energy (HZE) ions, as carbon (C), oxygen (O), neon (Ne), silicon (Si), calcium (Ca), and iron (Fe). The GCR energy spectra are very broad, ranging from 10 MeV/n to 50 GeV/n (Durante and Cucinotta, 2008). SPEs are composed of 90% protons, He ions, and a very small amount of HZE ions. About 21% of the dose equivalent to astronauts is constituted by HZE ions, 2% by Fe alone. HZE ions penetrate by many centimeters, producing along their track large ionizations’ clusters, differently from the more uniform energy depositions by low LET radiation (Simonsen et al., 2020). In fact, despite their concentration of around 1%, HZE ions, due to their high penetration and strong oxidizing power, are the most effective components to threaten human health (Norbury et al., 2016). Furthermore, for their capacity of inducing metabolism perturbations not only in the directly hit cells and in the nearby bystander ones but also in their progeny, HZE ions were demonstrated to induce long-lasting effects mimicking those of aging (Li et al., 2014).

Unlike what happens for low LET radiation, the HZE ion entrance dose is relatively low, reaching its maximum at the end of their tracks (Bragg peak) (Katz and Cucinotta, 1999). The ability of HZE ions to deposit the maximum dose at precise depth is exploited for the treatments of tumors. Along their track, HZE ions induce the generation of secondary radiations including photons, protons, neutrons, alpha particles, other heavy ions with lower velocity, and the energetic electrons d rays (Li et al., 2014). Due to their long range, the d rays may also hit cells far from the primary particle track, increasing by 30–40% the HZE ion dose (Yan et al., 2014). The concentration of radiolytic species induced in and around the particle track is very dense (Muroya et al., 2006; Cucinotta et al., 2011), causing extensive covalent modifications in targeted macromolecules. The persistence of DNA damage unrepaired, such as that induced by HZE ions, leads to the accumulation of DNA repair effectors ATM (ataxia-telangiectasia, mutated) and ATR (ATM and Rad3-related) at the sites of damage, originating DNA segments with chromatin alterations, hallmark of the cell’s senescence (Rodier et al., 2009). These modifications lead to changes in signaling events that may affect proteins and genes involved in the oxidative metabolism (Li et al., 2014).

## Physiological and Pathological Impact of Cosmic Rays

The radiation dose absorbed by astronauts during the long-duration space travels could exceed the admissible doses (Cucinotta, 2001), raising concern about the early and late health risks. The uncertainties in estimating space radiation risks have been recognized by several reports from the National Council on Radiation Protection and Measurements (NCRP) -Report 160 (Bolus, 2013). This uncertainty is largely due to the insufficient information on the radiobiology of HZE ions that produce both quantitative and qualitative differences in biological effects compared to gamma or X-rays, but until now, no sufficient human data are available and it was difficult to exclude the impact of hereditary traits (Cucinotta et al., 2015).

The exclusion of genetic factors was achieved in a study conducted on two identical twins, one sent for a year on the International Space Station, and the other remained on Earth (Garrett-Bakelman et al., 2019). Several physiological and biological parameters were compared including body mass, bone density, telomere length, genetic damage, transcriptional and metabolic alterations, immune response, oxidative metabolism, and a battery of tests for cognitive performances. Most of these did not show any significant differences or returned to baseline values after the return, while changes in the expression of some genes, genomic instability, and a decline of cognitive efficiency persisted even 6 months from the conclusion of the mission.

Long-term decline in learning recall was observed in patients undergoing brain radiotherapy without sparing the hippocampus (Gondi et al., 2013), whereas impairment of the hippocampus-dependent learning and memory was observed in rodents exposed to low doses of HZE ions (Britten et al., 2017). Significant loss of neuronal progenitor cells occurred within a few hours after low radiation doses, and the effects extended over time because the differentiation of the surviving neuronal progenitor cells depends on the amount of the delivered dose (Voloboueva and Giffard, 2011). Smart (2017) demonstrated that the neurodegeneration is mainly due to the loss of neurons, but other factors could contribute to the onset of cognitive deficits, as well as damage to the microcirculation, decreased metabolic activity, degeneration of synapsis and myelin, and glia proliferation. Impairment of hippocampal neurogenesis and alteration of microglial functions have recently been proposed as main causes of the aging-related cognitive decline (Chowen and Garcia-Segura, 2020). Krukowski et al. (2018b) demonstrated that the development of neurodegenerative disorders in aging mice was associated with a prolonged state of sterile inflammation, characterized by increased microglial cell number and phagocytic activity. This process was initiated by the complement components C1q, C3, and CR3, which regulate the microglial–synapse interactions. A single exposure of simulated cosmic rays was proven to induce in male mice long-term cognitive and behavioral impairment with increase in microgliosis and synapse loss in the hippocampus, but, remarkably, the female cohorts did not display any cognitive or behavioral deficits and no microglia changes (Krukowski et al., 2018a). The explanation is in the embryonic development which is distinct between males and females; in fact, estrogen priming in male increases the microglia cells’ reactivity, while in female, the hippocampal microglia cells are less reactive (Villa et al., 2018). A lower inflammatory response in female mice could explain the lack of hippocampal damage induced by HZE ions.

There is limited and sporadic epidemiologic data for long-term cardiovascular morbidity and mortality of United States astronauts. In a first study in astronauts who participated in the Apollo 11, 12, and 14–17 Moon missions, it was shown that heart attack was the second leading cause of death (Yan et al., 2014). Afterward, Delp et al. (2016) found that the astronauts who have traveled outside of the Earth’s protective magnetosphere showed a higher mortality rate due to cardiovascular diseases compared to non-flight astronauts and those who flew only low Earth orbit missions. In the same study, in mice treated with HZE ions was observed an altered response to acetylcholine (ACh) in vasodilation of muscle feed arteries, causing dangerous pressure failure. Low and moderate doses of HZE ions may increase the risk of cardiovascular diseases by altering DNA methylation and the expression of genes related to cardiovascular function (Koturbash et al., 2016) or by inducing a pro-inflammatory state (Hughson et al., 2018).

Cancer is another main risk faced by astronauts on long-term space exploration missions. The risk estimate cannot be inferred from the knowledge we have about terrestrial radiation. HZE ions in fact affect DNA by inducing, differently from low-LET radiation, multiple damaged sites and clustered DNA damage not easily repairable with the DDR system (Asaithamby and Chen, 2011). Kennedy et al. (2018) treated immortalized human bronchial epithelial cells with high LET ^56^Fe, ^28^Si, and low-LET X-rays to assess the respective methylation patterns. These radiations induced DNA methylation by distinct mechanisms involving different chromatin regions. ^56^Fe ions were found to affect the accessible chromatin region enriched of promoters and regulatory genes. These results were compared with those collected through the Genome Atlas Project, finding that the methylation of the CpG sites induced by ^56^Fe ions showed significant overlapping with the genes undergoing promoter hypermethylation in primary lung cancer. Thus, the DNA methylation pattern may be considered an enduring biomarker of exposure to HZE ions with the potential of long-term health impact, such as cancer.

## Involvement of the Mitochondria

In addition to regulating the production of ATP, mitochondria carry out multiple functions in the cell and their imbalance is associated with multisystem diseases, and many age-associated metabolic disorders are linked to defects of the mitochondrial respiratory chain (Alston et al., 2017). Notably, the brain and the heart, the organs most compromised in diseases related to mitochondrial dysregulation, are usually the most compromised also in astronauts after a long travel through space.

As reported in the previous paragraphs, the energy of high-LET radiation is deposited along a much smaller number of narrow tracks, therefore, when the HZE ions hit a mitochondrion, they are able to induce a strong oxidative imbalance, triggering persistent changes in the machines producing ROS and the activation of reactive nitrogen species (Datta et al., 2013). The first target of the radicals’ increase induced by HZE ions is the mitochondrial DNA (mtDNA) because it is particularly subjected to oxidative stress and then is close to the electron transport chain where mitochondrial ROS are produced. Already Harman (1972) had suggested for mtDNA a key role in the aging process, and mutated mtDNA has been found in neurons during normal aging and in neurodegenerative diseases in the cerebral cortex (Nissanka and Moraes, 2018).

The mtDNA repair machinery is very poor compared to the nuclear one; therefore, the organelle homeostasis is maintained by means of a continuous process of fusion/fission, which, among other things, leads to the elimination of damaged elements through mitophagy. It has been observed that in cells treated with ionizing radiation, the fusion/fission cycle is compromised, and this consequently induces the maintenance of non-functional mitochondria in which both the dysregulation of energy processes and the production of radicals continue (Jin et al., 2018). Also, in xeroderma pigmentosum and the Cockayne’s syndrome, mitochondria undergo an increase in mass and polarization, signs of imbalance of the remodeling process (Prates Mori and de Souza-Pinto, 2018). One hypothesis is that the malfunction of respiratory chain in radiation-damaged mitochondria together with nuclear signals not yet clarified could lead to a sharp decrease in NAD+ production, thus inhibiting mitophagy, a NAD + -dependent process (Fang et al., 2019).

Ultimately, damage to mtDNA can significantly affect cell function not only by decreasing the mitochondrial energy production but also by increasing the production of free radicals. In this way, the mitochondria continue to produce excess ROS which in turn participate in the formation of genomic damage, thus perpetuating the processes underlying senescence or alternatively of cell apoptosis. Increased oxidative stress and accelerated senescence signaling were observed in mice exposed to both γ and Fe^56^; importantly, HZE ions were proven to be more effective in inducing oxidative damage and accelerating aging compared to γ rays (Suman et al., 2013). Furthermore, high and persistent ROS levels could influence, through the bystander effect, the behavior of the neighboring cells, spreading damage in the tissues (Li et al., 2015). The key role of oxidative stress is highlighted by recent National Aeronautics and Space Administration (NASA) translational research guidelines. One of the four areas of interest indeed concerns the effect of space flights irradiation on the generation of reactive oxygen and nitrogen species capable of impact on astronauts’ health (Alwood et al., 2017).

But the increase in radicals does not seem to be the only factor capable of transferring the damage induced by HZE ions from the mitochondria to nuclear DNA. There also appears to be a two-way relationship between radiation-induced damage to nuclear DNA and mtDNA. On the one hand, it is known that the presence of persistent IR-dependent damage to nuclear DNA has an indirect effect on the mitochondrion’s functioning through an unclear mechanism. On the other hand, exposure to IR induces the transfer of mtDNA to the nucleus (Singh et al., 2017). This transfer is triggered by the induction of double-strand breaks in mtDNA, the fragments are then subsequently incorporated into nuclear genomic coding regions causing the induction of oncogenes and permanent changes in gene expression (Ricchetti et al., 2004).

## Conclusion

**FIGURE 1:**
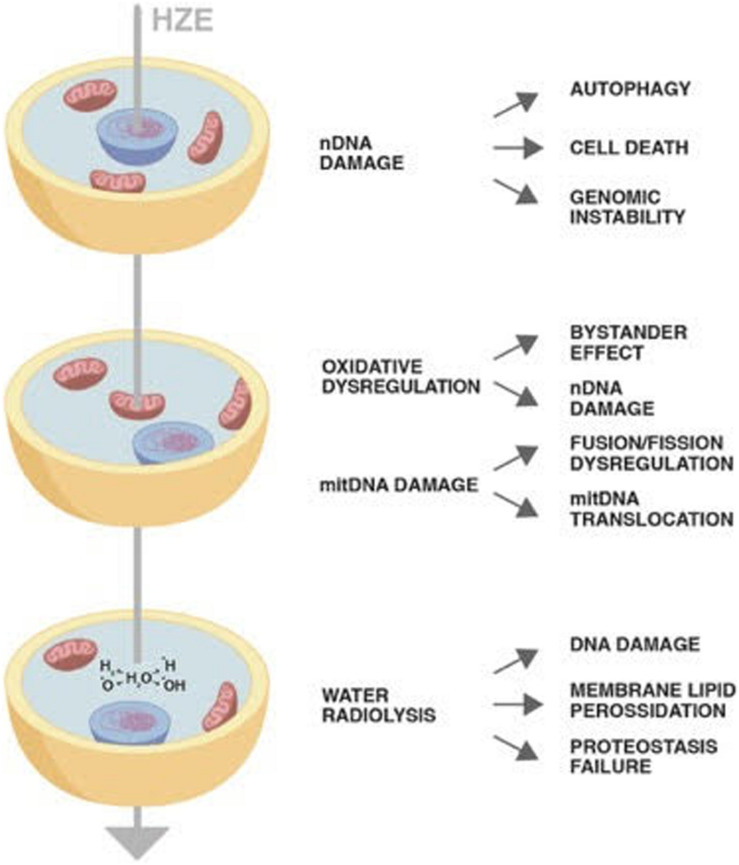
The diagram shows the impact of a High-Atomic number and High-Energy (HZE) ion on cells. The HZE ion can hit a column of cells depositing energy at different cell compartments: nucleus, mitochondria, and cytoplasm. The type and persistence of the effects triggered by the interaction of the particle with the biological matter may vary as indicated in the text.

Despite their concentration in the cosmic rays being around 1%, HZE ions due to their high penetration and strong oxidizing power have been proven to induce permanent damages through horizontal and vertical transmission. The direct or indirect damage through radiolysis of mitochondria has as a consequence not only failure of its metabolic role but also establishment of a persistent oxidative imbalance. Once damaged, mtDNA may insert in nuclear chromosomes perpetuating genomic instability (Figure 1). The cells bearing DNA damage that cannot be quickly repaired with the DDR system enter in apoptosis or in the quiescent state typical of senescence. Final result is a decrease in the tissue functionality as is occurring in aging. Therefore, cosmic rays would mimic the effect of aging, inducing a persistent state of sterile inflammation damaging DNA, proteins, and lipids the consequences of which are aging-related disease such as cardiovascular diseases, neurocognitive impairment, and increase of cancer occurrence.

## Author Contributions

All authors listed have made a substantial, direct and intellectual contribution to the work, and approved it for publication. In particular AG treated the part concerning the nature and effects of cosmic rays. SR the biological processes related to senescence and aging. FT the part related to mitochondria.

## Conflict of Interest

The authors declare that the research was conducted in the absence of any commercial or financial relationships that could be construed as a potential conflict of interest.
